# Complete chloroplast genome sequence of Coyote tobacco (*Nicotiana attenuata*, Solanaceae)

**DOI:** 10.1080/23802359.2017.1398611

**Published:** 2017-11-06

**Authors:** Péter Poczai, Ali Amiryousefi, Jaakko Hyvönen

**Affiliations:** a Finnish Museum of Natural History (Botany), University of Helsinki, Helsinki, Finland;; b Department Bioscience (Plant Biology), Viikki Plant Science Centre, University of Helsinki, Helsinki, Finland

**Keywords:** Chloroplast genome, *de novo* assembly, genome skimming, phylogenomics, plastid evolution

## Abstract

In this study, we announce the complete chloroplast genome sequence of *Nicotiana attenuata*. The genome sequence of 155,941 bp consists of two inverted repeat (IRa and IRb) regions of 25,438 bp each, a large single-copy (LSC) region of 86,513 bp and a small single-copy (SSC) region of 18,524 bp. The overall GC content is 37.9% and the GC contents of LSC, IRs, and SSC are 36%, 43.2%, and 32.1%, respectively. The plastome with 129 annotated unique genes includes 84 protein-coding genes, 37 tRNA genes, and 8 rRNA genes. Using the whole chloroplast genome sequences alignment of 16 Solanaceae species a phylogenetic hypothesis is presented validating the position of *N. attenuata* within Nicotianeae.

*Nicotiana attenuata* Torr. ex S.Watson is a diploid (2*n* = 2*x* = 24) wild tobacco native to the Great Basin region of the United States. This plant has adapted to an ecological niche defined by the post-fire environment, where soils tend to be nitrogen-rich and biotic stresses are highly dynamic (Nelson et al. 2012). During the last decade, large amounts of genomic, transcriptomic and metabolomic data have been generated for this plant which has provided new insights into how native plants interact with herbivores, pollinators and microbes (Brockmöller et al. 2017). Despite the availability of such large amounts of omics data the plastid genome of coyote tobacco has not been assembled. However, studies on the chloroplast genome could contribute greatly to our understanding of the molecular biology, physiology, and biochemistry of chloroplasts. Chloroplast DNA sequence data are a versatile tool for plant identification or barcoding and to enhance our understanding about phylogeny of plant species. Herein, we report the complete chloroplast sequence of *N. attenuata* to provide resources for further (phylo)genomic studies and chloroplast engineering.

Extraction of DNA was performed from 20 g of fresh leaves according to Shi et al. (2012) collected in Kaisaniemi Botanical Garden, University of Helsinki, Finland (60.1753; 24.9460; voucher P.Poczai 0012887). Illumina 2 × 150 bp paired-end libraries were prepared with the TruSeq DNA Sample kit followed by sequencing on a MiSeq platform (Illumina Inc., San Diego, CA). Raw reads were filtered with Trimmomatic (Bolger et al. 2014), and *de novo* assembly of the plastid genome was carried out with the Geneious R9.1.7 assembler platform (Kearse et al. 2012). We annotated the genome using Geneious and in-house scripts

The complete chloroplast genome of *N. attenuata* (GenBank accession no. MG182422) has a total length of 155,914 bp which is divided by two IR regions of 25,438 bp. The genome consists of 129 genes and has 37.9% overall GC content. The genes can be classified into 37 tRNA, eight rRNA and 84 protein-coding genes, from which seven tRNA, four rRNA and six protein-coding genes are duplicated in the IR.

Using the RAxMLv8.0 (Stamatakis 2014), we calculated the best scoring ML tree with 10,000 bootstrap replicates under GTR-GAMMA after running jModelTest2 (Darriba et al. 2012) including 15 representative species of Solanaceae and three outgroup species of Gentianales (Figure 1). The same matrix was analyzed also with parsimony as an optimality criterion using WinClada (Nixon 2002) and TNT (Goloboff et al. 2008). Prior to the analysis we used the WinClada command ‘Mop uninformative characters’ to exclude parsimony uninformative characters. This resulted in a matrix with 17,620 characters and due to its small size we were able to perform analyses using implicit enumeration of the TNT that ensures finding optimal tree(s).

**Figure 1.**
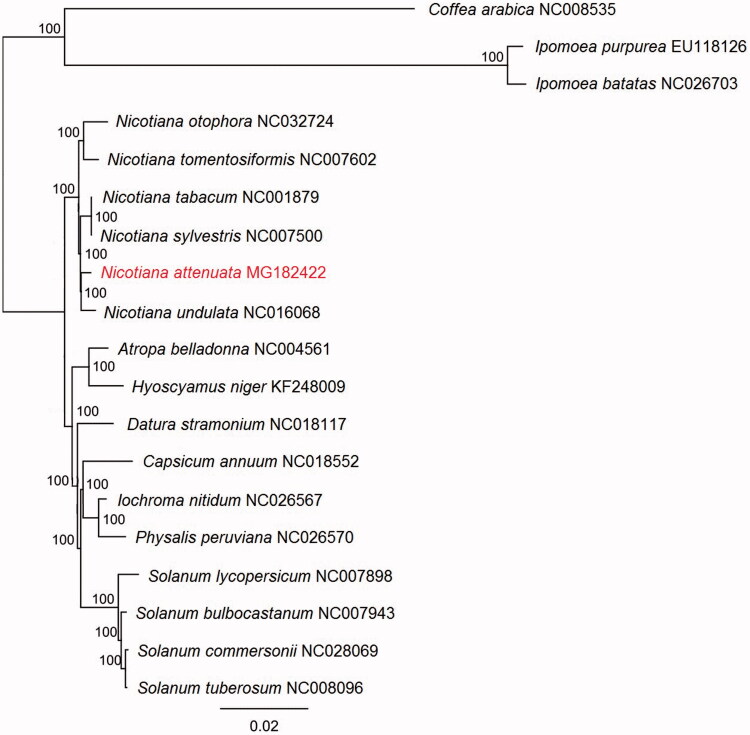
The ML tree of 18 selected chloroplast genome sequences and *Nicotiana attenuata*. The values on the node show the bootstraps of 10,000 replicates and scale is substitution per site.

We expect this sequence to help further taxonomic studies of the genus *Nicotiana* and provide additional genomic resources for chloroplast engineering.
